# Determination of Blood Glucose, Total Protein, Certain Minerals, and Triiodothyronine during Late Pregnancy and Postpartum Periods in Crossbred Dairy Cows

**DOI:** 10.1155/2021/6610362

**Published:** 2021-03-09

**Authors:** Shima Essameldin Mohammed, Faisal Omer Ahmad, Ehab A. M. Frah, Imadeldin Elfaki

**Affiliations:** ^1^Department of Biochemistry, Faculty of Veterinary Medicine, Sudan University of Science and Technology, Khartoum, Sudan; ^2^Department of Reproduction and Obstetrics, Faculty of Veterinary Medicine, University of Khartoum, Shambat, Sudan; ^3^Department of Statistics, Faculty of Science, University of Tabuk, Tabuk, Saudi Arabia; ^4^Department of Biochemistry, Faculty of Science, University of Tabuk, Tabuk, Saudi Arabia; ^5^Department of Biochemistry, Faculty of Veterinary Medicine, University of Khartoum, Shambat, Sudan

## Abstract

The late pregnancy (3^rd^ trimester) and the postpartum period (PPP) (calving date or day zero to day 45) are very critical periods for the fertility and production in dairy cows. This study was designed to investigate blood glucose, total protein (TP), calcium (Ca), phosphorus (P), magnesium (Mg), iron (Fe), and triiodothyronine (T3) during late pregnancy and the PPP. Twenty-seven apparently healthy multiparous crossbred dairy cows (Friesian × Kenana) were included in this study. The cows were randomly allocated into three groups: group A (*n* = 10), cows with late pregnancy, group B (*n* = 7), cows in the PPP, and group C (*n* = 10), nonpregnant cows as control. One-way ANOVA was used to analyze the data. The results of this study showed that blood glucose was higher in late pregnancy and the PPP than in nonpregnant cows. The TP was significantly lower in late pregnant cows than during the PPP and in nonpregnant cows. Ca, P, and Mg were not significantly different between periods. Serum Fe and T3 were significantly lower during the PPP than that in late pregnant and nonpregnant cows. The results can provide indications of the nutritional status of dairy cows and a diagnostic tool to avoid the metabolic disorders that may occur during late pregnancy and the PPP.

## 1. Introduction

The postpartum period (PPP) is important because of its effect on general health and subsequently reproductive performance of dairy cows. Cows develop serious metabolic and physiological changes during this critical period [1]. The vast majority of metabolic disorders occur during this time. During late pregnancy (3^rd^ trimester) and the PPP, the nutritional status of cows is important for the resumption of the ovarian activity [2, 3]. For instance, adequate level of glucose during late pregnancy and the early PPP in dairy cows is crucial for the growing fetus, reproductive performance, and milk production [4, 5]. Glucose regulates the concentrations of other blood metabolites [6], and low blood glucose in the PPP causes repeat breeding and reduces the conception rate (CR) [7, 8]. Many protein-related parameters are highly affected around parturition and may significantly impose to further reproductive performance [9]. Animals require minerals such as calcium (Ca), magnesium (Mg), and phosphorus (P) during late pregnancy and the postpartum period for growth, reproduction, and lactation, which often affect specific requirements and serve as catalytic components of enzymes or regulate several mechanism involved in pregnancy and lactation [10, 11]. Blood calcium level in late pregnancy and the early postpartum period is very important, and serum calcium concentrations reflect the ability of a cow to replace extracellular calcium lost in milk by withdrawing calcium from the bone and by increasing the efficiency of calcium absorption [12]. Most episodes of clinical hypocalcaemia in dairy cows occur in the first 24 hours after calving [13]. Magnesium, such as calcium, reduces neuromuscular irritability. A drop in blood Mg concentration results in spontaneous muscle contractions or tetany in dairy cows [14]. Hypomagnesemia occurs during the postpartum period in dairy cows [15, 16]. Parturition significantly influences the level of blood iron. Iron may be utilized in immunological challenges [17]; therefore, appropriate levels are necessary for ovarian rebound [18]. The activity of the thyroid gland is important in the peripartum period for optimum carbohydrates and lipids metabolism as well as lactation [19]. Furthermore, the role of thyroid hormones in the onset of postpartum period ovarian activity is well-established [20, 21]. T3 and T4 were lower in animals with inactive ovaries, and low levels of thyroid hormones may delay postpartum (PP) reproductive functions [20]. This study aimed to assess seven blood biochemical parameters (glucose, TP, Ca, P, Mg, Fe, and T3) during the late pregnancy and PPP because these periods are very important for the fertility and production in dairy cattle. The results can be an indicator for the cow health and a diagnostic tool to avoid the metabolic disorders that occur during the late pregnancy and PPP.

## 2. Materials and Methods

The Research Board (01/2020 on 01/03/2020), Faculty of Veterinary Medicine, University of Khartoum, approved this study. It was carried out in the University of Khartoum dairy farm during January to April 2020. Twenty-seven healthy multiparous crossbred dairy cows (Friesian × Kenana) were employed. The cows were under the semiclosed system and were milked manually twice a day in the morning (02 : 00 a.m.) and evening (02 : 00 p.m.). The age of cows between 4 and 8 years and their body condition scores (BCSs) vary between 2.5 and 3. The offered diet consists of roughages alfalfa (*Medicago sativa*) and abu 70 (*Sorghum bicolor*) and concentrates. The cows were allowed to graze daily from 07 : 00 a.m. to 10 : 00 a.m.  Table 1 shows the proximate analyses of roughages fed to the cows. Each dairy cow was fed rations individually twice a day at time of milking (10 kg/cow), which consist of 37% sorghum, 21% cotton seed cake, 40% wheat brand, and 2% sodium chloride [22]. The pregnancy diagnosis was performed by rectal palpation to verify pregnancy. Three blood samples were collected from cows in groups A and C with 7-day interval counting from calving day as day zero (i.e., on days 7, 15, and 21). Four blood samples were collected from cows in group B with 7-day intervals starting from calving date as day zero with the 7-day interval (i.e., on days 0, 7, 15, and 21).

### 2.1. Experimental Design

This study was designed to investigate seven blood biochemical metabolites (glucose, total protein, Ca, P, Mg, Fe, and T3) during late pregnancy and the PPP. Twenty-seven multiparous apparently healthy crossbred dairy cows (Friesian × Kenana) were employed for this study. The cows were divided randomly into three groups: group A (*n* = 10), cows with late pregnancy, group B (*n* = 7), cows in the postpartum period, and group C (*n* = 10), nonpregnant cows left as control. The blood parameters were assessed as mentioned above.

### 2.2. Collection of Blood Samples

Blood samples were collected from the jugular vein, using plastic disposable syringes. Ten milliliters of blood were collected, immediately 2 ml was transferred to a test tube containing the anticoagulant fluoride oxalate, and the sample was used for measurement of blood glucose. The rest of the blood samples were transferred to a plain tube and left to coagulate spontaneously at room temperature (28°C), then centrifuged at 2000 rpm for 10 min. Serum samples were separated and transferred to clean plastic vials and immediately frozen at −20°C for subsequent analyses.

### 2.3. Measurement of the Biochemical Parameters

The assessment of plasma glucose, serum total protein, Ca, P, Mg, and Fe was done by using commercial kits (BioSystems, S.A., Barcelona, Spain) by using A25-Biosystem-Random Access Analyzer, and it is a fully automated analyzer. The blood glucose level was determined immediately after blood sampling. The serum level of TT3 was determined using ST AIA-PACK TT3 (Cat. No. 0025282, Japan) on TOSOH AIA System Analyzers.

### 2.4. Statistical Analysis

Results were expressed as mean ± standard deviation (SD). The ANOVA test (one way) was used to investigate difference among the three animal groups. Significance level was considered at (*p* < 0.05). SPSS was used for this purpose.

## 3. Results

The findings of statistical analysis to our study are shown in Table 2. There was a high significant difference (*p* < 0.001) between the blood glucose concentration (mg/dl) during late pregnancy, postpartum period, and nonpregnancy. The serum total protein concentration (g/dl) was significantly different (*p* < 0.001) between these groups. There was no significant difference in serum calcium, phosphorous, and magnesium concentration during late pregnancy, postpartum period, and nonpregnant cows. The serum iron concentration (*µ*g/dl) is significantly different (*p* < 0.001) between the cows in late pregnancy, PPP, and nonpregnancy. The serum total triiodothyronine concentration (ng/dl) was significantly different (*p* < 0.001) between these groups.a, b: mean values within the same row with different superscripts are significantly different. The mean difference is significant at the 0.05 level. ^*∗∗*^, *p* < 0.01; ^*∗∗∗*^, *p* < 0.001; NS: not significant.

## 4. Discussion

Blood glucose controls the reproduction as it is the main modulator of blood hormones and metabolites [23]. It has been reported that in dairy cattle, adequate levels of blood glucose may be important for proper functioning of the ovaries and uterus and that blood glucose of 60 (mg/dL) is appropriate for the cow to get pregnant at first insemination [4]. In this study, blood glucose (mg/dL) levels were within normal range (40–60) [24], and that concentration of plasma glucose was higher during late pregnancy (67.89 ± 6.52) and in the PPP (65.88 ± 5.94) than in nonpregnant cows (57.40 ± 7.90). In the PPP, the serum glucose concentration was insignificantly lowered than in the pregnancy period, and this is probably due to the needs of lactose and fats for milk production [25]. This finding is perhaps consistent with a previous study reported that there is a reduction in blood glucose following the calving date. However, in this study, there is no statistical significant difference in blood glucose between late pregnancy and the postpartum period which is probably due to the small sample used (*n* = 27).

This study revealed that the level of serum total protein (TP, g/dl) during the late pregnancy (6.42 ± 0.55) was lower than in postpartum (6.93 ± 0.98) or nonpregnancy periods (7.18 ± 0.40) (Table 2). This result is in agreement with a recent study [26] which reported a decreased serum TP during late pregnancy. In the PPP, the level of serum TP was elevated compared to late pregnancy (Table 2). This is consistent with a previous study reported that serum TP levels were significantly affected from the physiological period and increased during lactation when compared to late gestation in dairy cows [27]. This change in protein concentration occurs because the cow in the gestation period experiences great metabolic stress [28].

Results showed that the serum Ca(mg/dl) of the cows is within normal range (2–10) [29, 30]. Results also indicated that the serum Ca level during late pregnancy (8.69 ± 0.50) was insignificantly different from the PPP (8.39 ± 0.91) and in nonpregnant cows (8.42 ± 1.19). This result may be in agreement with a study reported insignificant difference in calcium level between pregnant lactating and nonpregnant crossbred dairy cows [31]. Serum calcium level in this study was insignificantly lower during the postpartum period compared to late pregnancy (Table 2). This finding may be in agreement with a study reported that the serum Ca in dairy cows in early and midlactation were significantly lower than in late pregnancy [32]. Also, it may be in agreement with a study reported lower levels of serum Ca in cows immediately after calving and up to fifteen days from calving [33]. However, in the current study, there was no statistically significant difference in Ca levels between the 3 periods (Table 2), and this is perhaps because of the small sample size used (*n* = 27).

Results showed that the level of serum P (mg/dl) during late pregnancy (6.44 ± 1.19) is insignificantly higher than the PPP (6.33 ± 1.38) and in nonpregnant cows (6.05 ± 1.57). This finding is consistent with the results of previous studies conducted in crossbred dairy cows [29, 32, 34]. Again a significant difference was not seen probably because of the small sample size used in this study.

Results indicated that serum level of Mg (mg/dl) is within the normal range (2–2.4) [29, 30] and that the Mg levels are lowest during late pregnancy (2.10 ± 0.30) than in the PPP (2.15 ± 0.38) and nonpregnant cows (2.38 ± 0.25). This result is in agreement with a recent study conducted also in Sudanese dairy cows [29]. This may be due to the increased usage of Mg by dairy cow during the periparturient period [14].

Results showed that the level of serum Fe (*µ*g/dl) is lower during the PPP (124.76 ± 33.50) than in the late pregnancy (158.51 ± 36.50) and nonpregnant cows (142.93 ± 40.07); however, they were within normal range [35, 36]. This result is in agreement with a recent study reported that from the day of calving, concentration of Fe is decreased [18]. Also, the current result is consistent with an early study showed that serum Fe and some Fe binding measures are decreased at calving in crossbred Friesian cattle [37]. It has been reported that parturition may significantly decrease the concentration of serum Fe because of immunological reactions and ovarian activity [18]. Therefore, the blood levels of Fe should be monitored carefully in this period.

In the present study, the serum levels of T3 (ng/dl) are lower during the PPP (4.27 ± 0.85) than in late pregnancy (4.96 ± 0.64) and nonpregnant cows (5.58 ± 0.97). This result is in partial agreement with previous studies reported that there was a reduction of serum T3 and T4 in postcalving compared with the precalving periods [38, 39]. This reduction in T3 level may be due to the conditions of negative energy balance that is characterized by increased mobilization of nonesterified fatty from body reserves in the postpartum periods [39]. The thyroid hormone concentrations are regarded as an indicator of energy balance in the dairy cows [39]. The limitations of this study include the relatively small sample size employed, and the effects of the age and body condition score (BCS) have never been considered. Future well-designed studies with larger sample sizes considering the effects of age and BCS are recommended.

## 5. Conclusion

This study concluded that the late stage of pregnancy and PPP have influenced blood biochemical metabolites and minerals. It was observed that during these periods, there are dynamic changes in the blood concentration of glucose, TP, Fe, and T3. The current result revealed that lower concentration of these parameters could be used to evaluate the cow's health status.

### 5.1. Recommendation

Since the late pregnancy and the PPP are critical for the reproductive efficiency and production in dairy cows, the regular checkup of blood biochemical metabolites and minerals in these periods is very important. Accordingly, supplementation of a balanced ration and minerals are recommended. Moreover, during the late pregnancy, the lactating cows should be dried off from 4 to 6 weeks prior to calving date.

## Figures and Tables

**Table 1:** Analyses of the roughages used to feed the dairy cows.

Ingredients	Alfalfa (*Medicago sativa*)	Abu 70 *(Sorghum bicolor*)
Dry matter (%)	23	28
Crude protein (%)	3.6	3.1
Crude fibre (%)	6.7	8.7
Fat (%)	1.75	1.33
Ash (%)	2.6	4.1

**Table 2:** Blood glucose, total protein (TP), calcium (Ca), phosphorus P, magnesium (Mg), iron (Fe), and triiodothyronine (T3) during late pregnancy and postpartum periods and in nonpregnant crossbred dairy cows. Results are expressed as mean ± SD.

Group				
Parameters	Late pregnancy	Postpartum period (PPP)	Nonpregnant	*p* value
Glucose, mg/dl	67.89 ± 6.52^a^	65.88 ± 5.94^a^	57.40 ± 7.90^b^	^*∗∗∗*^
Total protein (TP), g/dl	6.42 ± 0.55^a^	6.93 ± 0.98^b^	7.18 ± 0.40^b^	^*∗∗*^
Ca, mg/dl	8.69 ± 0.50	8.39 ± 0.91	8.42 ± 1.19	NS
P, mg/dl	6.44 ± 1.19	6.33 ± 1.38	6.05 ± 1.57	NS
Mg, mg/dl	2.10 ± 0.30^b^	2.15 ± 0.38^ab^	2.38 ± 0.25^a^	NS
Fe, *µ*g/dl	158.51 ± 36.50^a^	124.76 ± 33.50^b^	142.93 ± 40.07^ab^	^*∗∗*^
T3, ng/dl	4.96 ± 0.64^b^	4.27 ± 0.85^a^	5.58 ± 0.97^b^	^*∗∗*^

## Data Availability

The data used to support the findings of this study are included in the manuscript.
